# Psychotic episode associated with autoimmune limbic encephalitis in a patient treated with checkpoint inhibitors: a case report

**DOI:** 10.1192/j.eurpsy.2025.2035

**Published:** 2025-08-26

**Authors:** P. Sánchez Díez, C. Blanes Morell, J. Torres Cortés

**Affiliations:** 1 Psychiatry, Ramón y Cajal Hospital, Madrid, Spain

## Abstract

**Introduction:**

Immune checkpoint inhibitors are being used in patients with advanced malignancies. Although it can effectively treat tumors, 30–60% of patients could experience immune-related adverse events such as encephalitis with antibodies against the NMDA receptor.

We present a case of a 57-years-old man with no prior mental health history who was diagnosed of kidney cancer and received treatment with checkpoint inhibitors. He developed incoherent speech, visual hallucinations, delusional megalomania, disorientation, sleepiness and a low-grade fever of 37.7ºC. He was admitted in Neurology unit and diagnosed of autoimmune limbic encephalitis in a patient treated with checkpoint inhibitors.

**Objectives:**

To describe a case of a psychotic episode associated with autoimmune limbic encephalitis in a patient treated with checkpoint inhibitors.

**Methods:**

Clinical assesment and bibliographic review of pertinent literature.

**Results:**

During his admission in Neurology ward, the patient was suspicious, inattentive, aggressive with healthcare staff and he developed incoherent speech with visual hallucinations.

MRI suggested bilateral limbic encephalitis and the antibody test in cerebrospinal fluid were positive for NMDA receptor.

The psychotic episode was treated with olanzapine up to 20 milligrams and the limbic encephalitis with rituximab with a good response.

**Conclusions:**

The case presented is consistent with other reports of psychotic symptoms and development of encephalitis associated with antibodies against the NMDA receptor.

The diagnosis of anti-NMDAR encephalitis is usually delayed.

The differential diagnosis should be established with primary psychiatric disorders.

**Disclosure of Interest:**

None Declared

